# Supplementary material for: Genotype-Phenotype Correlations in 208 Individuals with Coffin-Siris Syndrome
Source: Genes (Basel). 2021 Jun 19;12(6):937. doi: 10.3390/genes12060937 (PMC8233770; doi:10.3390/genes12060937)
Supplement: Supplementary file 1 [file genes-12-00937-s001.zip › genes-1250029-supplementary.pdf]

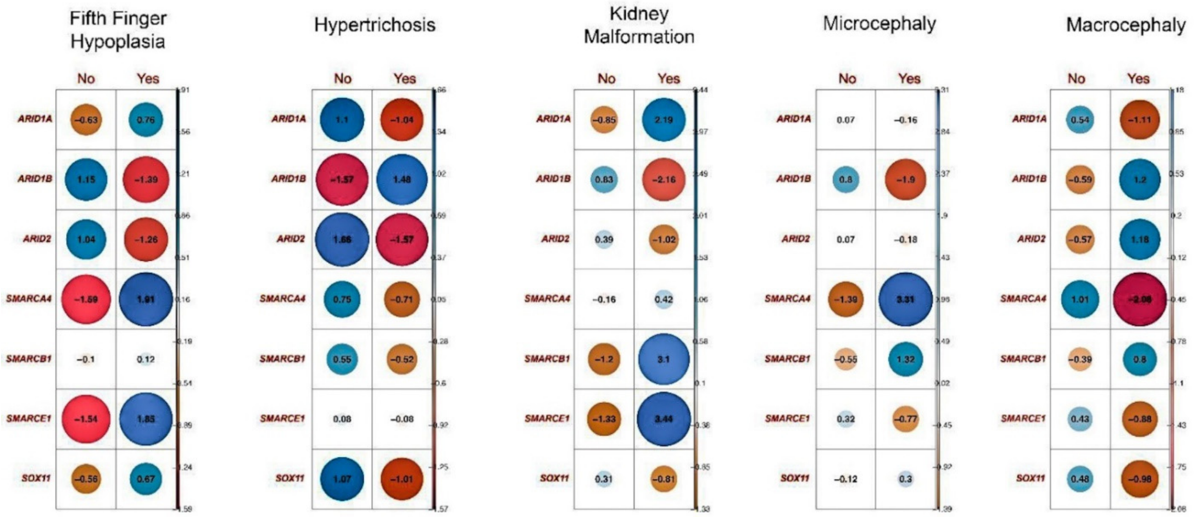

Supplemental Figure S1. Adjusted Pearson residuals for five binary phenotypes in individuals with variants in the BAF complex.

Supplemental Figure S1 visualizes the adjusted Pearson residuals for the five binary phenotypes with $p < 0.05$ from Chi-square analyses. The size and color scale of each circle indicate the magnitude of the residual, with a blue color scale used for residual values > 0 and a red color scale used for residuals < 0. For each phenotype, the genotype group is displayed along the vertical axis, while the binary response (yes = phenotype present, no = phenotype absent) is displayed along the horizontal
